# Changes in Antibiotic Resistance of *Acinetobacter baumannii* and *Pseudomonas aeruginosa* Clinical Isolates in a Multi-Profile Hospital in Years 2017–2022 in Wroclaw, Poland

**DOI:** 10.3390/jcm12155020

**Published:** 2023-07-30

**Authors:** Beata Mączyńska, Agnieszka Jama-Kmiecik, Jolanta Sarowska, Krystyna Woronowicz, Irena Choroszy-Król, Daniel Piątek, Magdalena Frej-Mądrzak

**Affiliations:** 1 Department of Pharmaceutical Microbiology and Parasitology, Faculty of Pharmacy, Medical University, 50-367 Wroclaw, Poland; 2 Department of Basic Sciences, Faculty of Health Sciences, Medical University, 50-367 Wroclaw, Poland; 3 Medical Laboratory Synevo, Fieldorfa 2, 50-049 Wroclaw, Poland; 4 Lower Silesian T. Marciniak Specialist Hospital-Center for Emergency Medicine, 54-049 Wroclaw, Poland

**Keywords:** multidrug resistance strains, carbapenems, non-fermenting bacilli

## Abstract

In recent years, we have witnessed increasing drug resistance among bacteria, which is associated with the use and availability of an increasing number of broad-spectrum antimicrobials, as well as with their irrational and excessive use. The present study aims to analyze changes in the drug resistance of Gram-negative *Pseudomonas aeruginosa* and *Acinetobacter baumannii*, isolated from infections in a multi-profile hospital over a five-year period (from 2017 to 2022). Among the practical results of the evaluation of these data will be the possibility to determine changes in susceptibility to the antibiotics used in the hospital. This, in turn, will help propose new therapeutic options, especially for empirical therapy, which is essential in severe infections. Analysis of the use of different antibiotic groups has made it possible to identify the causes of increasing resistance in the analyzed Gram-negative bacilli. The highest antibiotic use was observed in the hospital between 2020 and 2022, most probably due to the COVID-19 pandemic and the higher number of patients in severe condition requiring hospitalization. Unfortunately, during the period analyzed, the number of multi-resistant strains of *A. baumannii* was successively increasing; this seems to be related to the increased use, especially during the pandemic period, of broad-spectrum antibiotics, mainly penicillins with inhibitors, third-generation cephalosporins and carbapenems.

## 1. Introduction

In order to gather information on the prevention and control of infections and infectious diseases, the tasks and management of disease outbreaks or threats of disease outbreaks are defined, including the definition of an infectious disease as any disease caused by a pathogenic biological agent, and an alarming agent as a pathogenic agent with high virulence or resistance. Antimicrobial resistance could result in the global loss of 10 million lives per year by 2050 [1]. Strains showing acquired resistance to at least one antibiotic in a minimum of three significant groups (MDR) are of greatest concern. There are also XDR and PDR strains, which means resistance to at least one antibiotic of each group and insensitivity to all available therapeutics, respectively [2]. Apart from *Staphylococcus aureus*, *Enterococcus* spp., *Escherichia coli*, *Klebsiella pneumoniae* and *Streptococcus pneumoniae*, the most common bacterial carriers of resistance genes with high clinical relevance are *Acinetobacter baumannii* (*A. baumannii*) and *Pseudomonas aeruginosa* (*P. aeruginosa*), and it is these bacteria and their resistance that were analyzed in this study [3].

*P. aeruginosa* are the etiologic agent of many hospital-acquired infections, especially in elderly or immunocompromised patients. It is estimated that *P. aeruginosa* is the etiologic agent of 10–20% of infections among hospitalized patients [4]. Increasing resistance of this pathogen to commonly used chemotherapeutics has become a major problem. *A. baumannii* and *P. aeruginosa* show natural resistance to penicillin G, aminopenicillins, first- and second-generation cephalosporins, macrolides, tetracyclines, chloramphenicol, quinolones, sulfonamides and trimethoprim. Such a wide lack of sensitivity of *Pseudomonas* is mainly due to poor outer membrane permeability, high activity of membrane pumps and the production of enzymes capable of hydrolyzing antibiotics. Decreased outer membrane permeability caused by loss of the porin protein OprD leads to increasing resistance to carbapenems. The active efflux mechanism, on the other hand, ensures the cell’s safety when drug molecules enter the cell. The ABC or MDR pumps transport drugs from cell, making it easier for bacteria to survive antimicrobial therapy. These resistance mechanisms make these bacilli a very difficult pathogen to eradicate [5]. An additional feature of *P. aeruginosa* is the presence in its chromosome of a gene encoding β-lactamase AmpC, whose production depends on the presence of a drug in the environment. The effect of the mutation can be constitutive production of this enzyme, without induction, eventually leading to widespread resistance to first-, second- and third-generation cephalosporins, penicillins (including those with inhibitors) and aztreonam. Of greatest clinical importance, however, are traits that *P. aeruginosa* acquires through horizontal gene transfer, most commonly through genes encoding β-lactamases. Many of these enzymes function as penicillinases that remain sensitive to carbapenems and oxyimino-cephalosporins, with PSE-1 being the most common among clinical strains. Mutations in these genes have led to the development of a new type of enzyme capable of degrading oxyimino-β-lactams as well. The first characterized and also the most common ESBL in these bacteria was the PER-1-type enzyme, which is characterized by intensive hydrolysis of ceftazidime [6].

Similar to *P. aeruginosa*, *A. baumannii*, due to their ability to survive in a wide range of environmental conditions (high resistance to desiccation and disinfectants), very often populate hospital wards, causing opportunistic infections, especially in critically ill and long-term hospitalized patients. Long-term hospital stays, ICU treatment, mechanical ventilation (ventilator-associated pneumonia, so-called VAP), broad-spectrum antibiotic therapy and recent surgical interventions are considered predisposing factors for infection. Like *P. aeruginosa*, *A. baumannii* is responsible for serious nosocomial infections, such as pneumonia, in patients on artificial ventilation and is also a cause of urinary tract infections, meningitis, wound infections and sepsis. The bacteria exhibit strong adhesion properties and multidrug resistance [7]. Infections caused by MDR bacteria in seriously ill patients in ICU wards cause a high risk of death (from 26% to 68%) [8]. The results of the study also confirmed the correlation between the resistance of these bacteria isolated from infections and mortality among patients, as well as between the type of infection and a significant increase in hospitalization time [9]. Strains of *A. baumannii* species show natural resistance to ampicillin, amoxicillin (also with an inhibitor), ceftazoline, cefotaxime, ceftriaxone, ertapenem, trimethoprim and fosfomycin. Sulbactam, on the other hand, is the only inhibitor that shows activity against these bacteria [10]. The production of AmpC-type (ADC) and OXA-51-like β-lactamases, as well as the presence of membrane pumps and the occurrence of insertion sequences, have been cited as reasons for such widespread natural resistance [11]. In addition to natural resistance, the bacteria also develop acquired resistance. The increased loss of sensitivity to carbapenems is a worrying phenomenon. The above is most often caused by the overlap of several resistance mechanisms: production of carbapenemases, reduction of outer membrane permeability and the efflux mechanism [12]. Regular analysis of changes in drug resistance is a very good tool for making changes in empirical therapy procedures and improving the effectiveness of antibiotic therapy. The main elements of antibiotic policy at the hospital level include properly administered empiric therapy, promotion of targeted therapy, perioperative antibiotic prophylaxis and a properly constructed hospital formulary [13]. Antibiotic consumption and the cost of antibiotic therapy are therefore subject to constant control through monitoring the drug resistance of isolated microorganisms and effective antibacterial therapy for patients, leading to a reduction in the number of nosocomial infections and an improvement in the epidemiological situation, thereby reducing the cost of antibiotic therapy [14]. 

Our study aimed to assess the drug resistance of *P. aeruginosa* and *A. baumannii* isolated from infections in a multi-profile hospital over a period of 6 years (from 2017 to 2022) and to determine trends among resistance mechanisms in these microorganisms in relation to the level of antibiotic consumption during the analyzed period.

## 2. Materials and Methods

### 2.1. Bacterial Strains

All the analyzed data came from the information and materials of the multi-profile hospital in Wroclaw and cover the period from 1 January 2017 to 31 December 2022. The materials were obtained from patients with nosocomial infections. In order to observe changes in the drug susceptibility of bacteria isolated from patients, 2 species of great importance from the point of view of drug resistance of pathogens were selected: *P. aeruginosa* and *A. baumannii*. These strains were isolated from nosocomial infections, mainly from pneumonia in patients undergoing artificial ventilation, urinary tract infections, surgical site infections and primary bloodstream infections. The number of strains between 2017 and 2022 is shown in Table 1. With the exception of 2021, the frequency of hospital infections caused by *P. aeruginosa* was significantly higher than that caused by *A. baumannii*.

Depending on the strain, resistance to specific selected antibiotics was analyzed.

### 2.2. Microbiological Assays

#### 2.2.1. Automated Systems

The study was conducted according to the scheme: from the identification of strains through drug resistance analysis to the detection of carbapenemases by enzymatic and disk diffusion methods [15]. To identify microorganisms, strains were isolated from patient materials on appropriate microbiological media. Identification was made by evaluating colony morphology on plates and then using the VITEK^®^2 system (bioMerieux, Crappone, France). This system allows not only for confirmation of species affiliation, but also for the performance of antibiograms. This uses a bacterial suspension with a density of 0.5 on the McFarland scale and the corresponding identification and antibiogram cards. The corresponding VITEK^®^2 identification and antibiogram cards (AST-N331, GN ID), for the different microorganisms were placed in a VITEK^®^2 (bioMerieux, France), and a computer program identified and assessed the drug susceptibility of the strains. To assess drug susceptibility with the VITEK2 system, an antibiogram card for Gram-negative bacteria was used—VITEK2 AST-331 (amikacin, ampicillin/sulbactam, aztreonam, cefepime, ceftazidime, ciprofloxacin, colistin, gentamicin, imipenem, levofloxacin, meropenem, piperacillin, piperacillin/tazobactam, ticarcillin/clavulanic acid, tobramycin, trimethoprim/sulfamethaxazole)—a set of 16 antibiotics (Table 2). The use of this system made it possible to determine MIC values. With some antibiotics absent from the VITEK panels, bacterial susceptibility was also tested using the antibiotic diffusion method (E-tests). The results obtained were interpreted in accordance with EUCAST recommendations [16,17].

#### 2.2.2. The Disk Diffusion Method

A disk diffusion method was used to identify extended-spectrum β-lactamases (ESBLs). Disks with ceftazidime and cefotaxime were used, arranged at a distance of 2 cm (between the centers) from the disk with amoxicillin-clavulanic acid. A positive test result was recorded when there was a marked enlargement of the zone of inhibition around the disk with ceftazidime or cefotaxime (cefpodoxime, aztreonam) on the side of the disk containing clavulanic acid. This enlargement can take very different shapes [18,19]. 

In turn, disk methods were used to identify the type of carbapenemases. For the phenotypic test detecting KPC carbapenemases, a 10-μg meropenem disk and a 10-μg meropenem disk soaked in boronic acid were used, maintaining a minimum distance of 3 cm between them, and the plate was incubated at 35 °C for 18 h. In *Pseudomonas* spp. and *Acinetobacter* spp. a difference in zones of inhibition of ≥7 mm is considered a positive result. For the detection of MBL-class carbapenemases, sterile disks soaked in EDTA solution and disks with 30 μg ceftazidime and 10 μg imipenem were used, maintaining a 2 cm gap between them [20]. A positive result was considered to be an enlargement of the zone of inhibition around the disk with CAZ30 and/or IMP10 toward the disk with EDTA. Detection of OXA-48 carbapenemases was possible using a disk with 30 μg temocillin [18,19]. A reduction in the zone of inhibition around the TEM30 disk ≤10 mm was then observed.

#### 2.2.3. Enzyme and Immunochromatographic Tests

The Carba NP enzyme assay (Argenta, Ferrara, Italy) allows detection of carbapenemases in *P. aeruginosa* and *A. baumannii*, but without determining their type. The test is based on the hydrolysis of imipenem by carbapenemases released from bacterial cell lysates suspended in buffer containing phenol red. As a result of the hydrolysis of imipenem, there is a change in the pH of the reaction environment (acidification), which is observed visually as a change in the color of phenol red to yellow or orange The difference in color in the test and control tubes indicates a positive test (carbapenemase production). The test was performed according to the recommendations of KORLD [21].

Resist O.O.K.N.V. immunochromatographic assays were also used to detect carbapenem hydrolyzing enzymes in the test strains. (Coris Bioconcept, Gembloux, Belgium). They allow the detection of carbapenemases KPC, OXA-163, OXA-48, NDM and VIM in Carbapenemase-producing organisms (CPOs), including *Pseudomonas aeruginosa* and *Acinetobacter baumannii*, which show resistance not only to beta-lactams and other groups of antibiotics, but also to carbapenems [21].

### 2.3. Statistical Analysis

The variables were expressed as a frequency: as an absolute value and as a percentage. Chi-square tests were used to compare categorical variables and Cochran–Armitage test for trends. Two-sided *p*-value < 0.05 indicated statistical significance. Statistical analyses were performed using Pipe-Friendly Framework for Basic Statistical Tests [R package ver. 0.7.0.]

## 3. Results

### 3.1. The Place of Isolate Pseudomonas aeruginosa and Acinetobacter baumanii Strains in Infections Detected at the Hospital during Period of 2017–2022

Non-fermenting bacilli are not frequently isolated microorganisms in the analyzed hospital. These strains rank fifth and sixth in frequency of isolation after *E. coli*, *K. pneumoniae*, *S. aureus* and *E. faecium* (Table 3). However, they are important pathogens causing severe infections, e.g., in the ICU.

The percentage of *Pseudomonas aeruginosa* strains in relation to other etiological agents of infection, shows a relatively constant level (7.4–6.2%) with a downward trend in recent years (Table 4). In the case of *Acinetobacter baumanii*, there was a clear increase in the percentage of strains in 2021, during the pandemic period (6.9%). However, in the following year, the percentage of these microorganisms decreased again (4.2%).

### 3.2. Antibiotic Resistance of P. aeruginosa Strains in 2017–2022

The analysis of changes in the resistance of non-fermenting *P. aeruginosa* included groups of antibiotics important from the point of view of therapy, such as cephalosporins, carbapenems, aminoglycosides and quinolones. Changes in the sensitivity of these bacteria to penicillins were not analyzed due to their natural resistance to these antibiotics.

**Figure 1:**
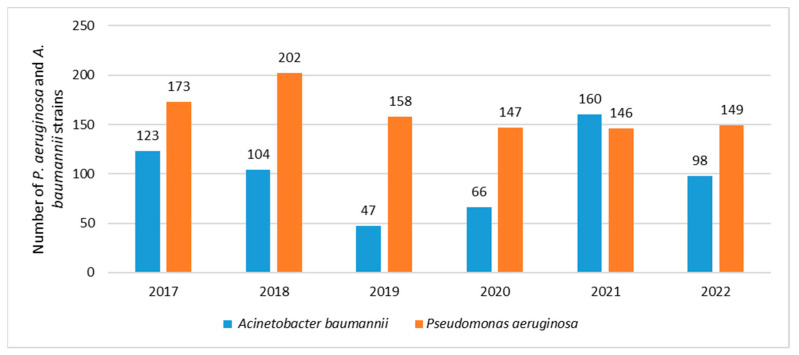
Comparison of the number of *P. aeruginosa* and *A. baumannii* strains isolated from infections between 2017 and 2022.

Between 2017 and 2022, the number of *P. aeruginosa* strains was at a similar level, ranging from 146 in 2021 to 202 in 2018 (Figure 1). In the case of *A. baumannii*, a downward trend was noted between 2018 and 2020. In subsequent years, the number of *Acinetobacter* strains increased from 47 in 2019 to 160 in 2021. However, after the pandemic, another significant decrease in their number was noted in 2022. (Figure 1).

**Figure 2:**
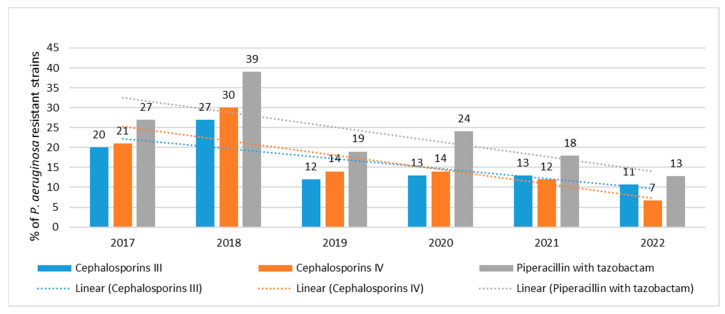
Percentage of *P. aeruginosa* strains resistant to cephalosporins and piperacillin with tazobactam.

Resistance of *P. aeruginosa* to cephalosporins remained low, ranging from 11% (*n* = 16; 2022) to 27% (*n* = 55, 2018) for ceftazidime and from 7% (*n* = 10; 2022) to 30% (*n* = 61; 2018) for cefepime (Figure 2). In contrast, sensitivity to piperacillin with tazobactam was slightly lower, with the percentage of resistant strains ranging from 13% (*n* = 19; 2022) to 39% (*n* = 79; 2018) (Figure 2). Resistance to third- and fourth-generation cephalosporins and piperacillin with tazobactam was the highest in 2018, with the number of strains resistant to these three groups of antibiotics in the recent year (2022) by far the lowest compared to previous years. Resistance trends to cephalosporins and piperacillin with tazobactam are highly statistically significant *p* < 0.001.

**Figure 3:**
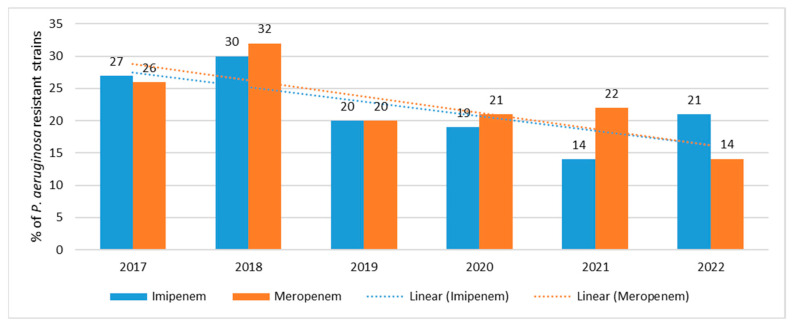
Percentage of *P. aeruginosa* strains resistant to carbapenems.

The percentage of carbapenem-resistant strains has remained relatively stable and fairly low over the past 6 years, ranging from 14% (*n* = 21; 2021) to 30% (*n* = 61; 2018) for imipenem and from 14% (*n* = 21; 2022) to 32% (*n* = 65; 2018) for meropenem (Figure 3). The highest number of resistant strains was isolated from hospital-acquired infections in 2018, while the lowest was in 2022. Resistance trends for carbapenems are highly statistically significant *p* < 0.001.

**Figure 4:**
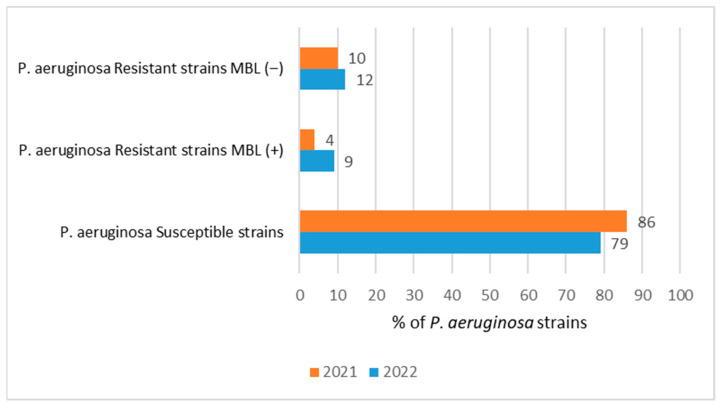
Comparison of the percentage of *P. aeruginosa* strains sensitive and resistant to carbapenems and strains producing MBL (+) carbapenemases in 2021 and 2022.

Figure 4 shows that the highest percentage (86%) of *P. aeruginosa* strains susceptible to carbapenems was recorded in 2021. At the same time, among the strains resistant to carbapenems, only 4% produced carbapenemases (MBL type), the remaining majority (10%) had a transport mechanism of resistance (efflux or porin mutations in the cell wall). In 2022, the situation changed somewhat. *P. aeruginosa* strains sensitive to carbapenems prevailed, although the percentage decreased slightly (79%), at the expense of an increase in strains producing MBL carbapenemases (9%). The percentage of carbapenem-resistant strains that did not produce metallo-β-lactamases (MBL (−)) and whose resistance was due to a mechanism other than enzymatic was 12%, a figure similar to the previous year’s value (10%) (Figure 4).

**Figure 5:**
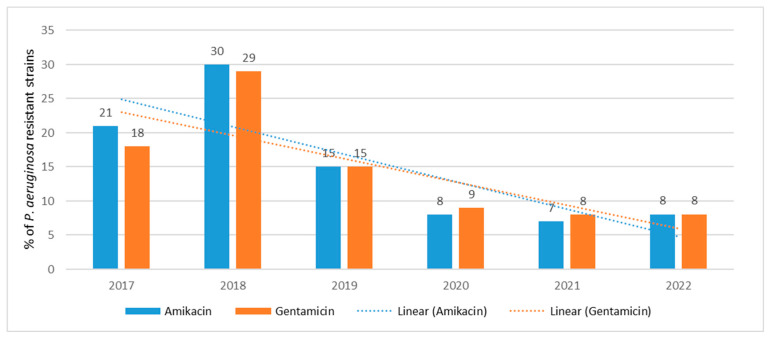
Percentage of *P. aeruginosa* strains resistant to aminoglycosides.

Resistance in *P. aeruginosa* over the past 6 years has ranged between 7% (*n* = 10; 2021) and 30% (*n* = 61; 2018) for amikacin and between 8% (*n* = 12; 2021) and 29% (*n* = 59; 2018) for gentamicin (Figure 5). The most resistant strains were isolated in 2018, and a gradual increase in the susceptibility of these bacteria to aminoglycosides was observed starting in 2019. Aminoglycoside resistance trends are highly statistically significant *p* < 0.001.

**Figure 6:**
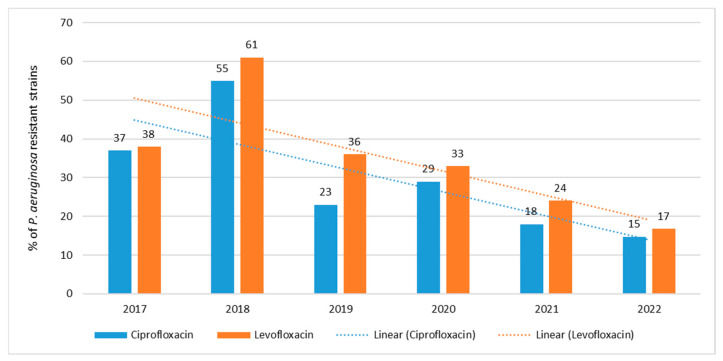
Percentage of *P. aeruginosa* strains resistant to quinolones.

The percentage of quinolone-resistant strains ranged from 15% (*n* = 22; 2022) to 55% (*n* = 111; 2018) for ciprofloxacin and from 17% (*n* = 25; 2022) to 61% (*n* = 123; 2018) for levofloxacin (Figure 6). The highest percentage of resistant strains was isolated in 2018, while the highest percentage of susceptible strains was observed in 2022. Quinolone resistance trends are highly statistically significant *p* < 0.001.

### 3.3. Antibiotic Resistance of A. baumannii Strains in 2017–2022

In purpose of observing the evolution of drug susceptibility of *A. baumannii* bacilli, the resistance of these strains to such antibiotic groups as carbapenems, aminoglycosides and quinolones was analyzed.

**Figure 7:**
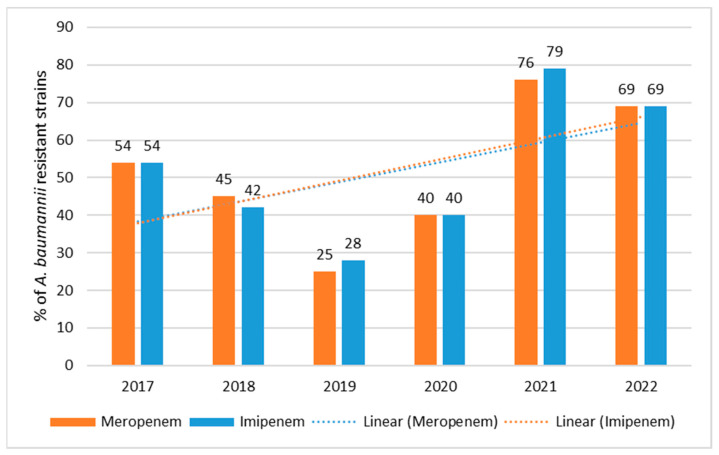
Percentage of *A. baumannii* strains resistant to carbapenems between 2017 and 2022.

The percentage of carbapenem-resistant strains ranged from 28% (*n* = 13; 2019) to 79% (*n* = 126; 2021) for imipenem and from 25% (*n* = 12; 2019) to 76% (*n* = 122; 2021) for meropenem (Figure 7). The least resistant strains were isolated in 2019 and the most resistant in 2021. Resistance trends for carbapenems are highly statistically significant *p* < 0.001.

**Figure 8:**
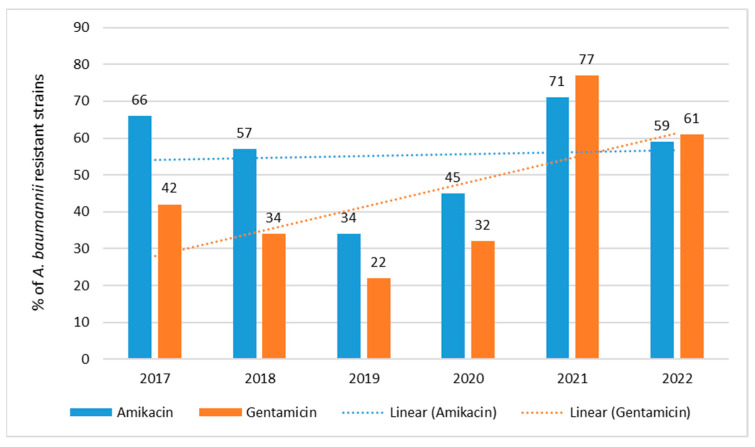
Percentage of *A. baumannii* strains resistant to aminoglycosides in 2017–2022.

The percentage of aminoglycoside-resistant strains was quite high, ranging from 34% (*n* = 16; 2019) to 71% (*n* = 114; 2021) for amikacin and from 22% (*n* = 10; 2019) to 77% (*n* = 123; 2021) for gentamicin (Figure 8). The highest resistance was observed in 2021 and the lowest in 2019. *A. baumannii* showed higher resistance to aminoglycoside antibiotics than *P. aeruginosa* strains (Figure 5 and Figure 8). The trend of gentamicin resistance is highly statistically significant *p* < 0.001, while the trend for amikacin lacks statistical significance.

**Figure 9:**
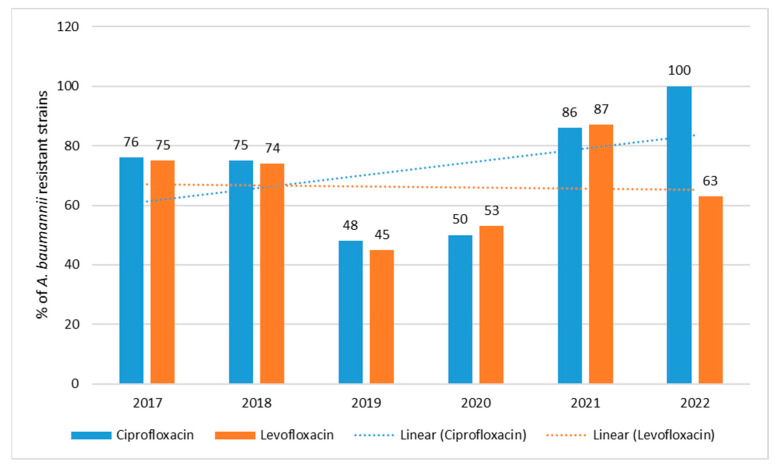
Percentage of *A. baumannii* strains resistant to quinolones in 2017–2022.

Among *A. baumannii*, quinolone resistance was observed, which was higher than that of *P. aeruginosa* (Figure 6 and Figure 9). The lowest percentage of resistant strains was observed in 2019–2020 and ranged from 48% (*n* = 23; 2019) to 50% (*n* = 10; 2019) for ciprofloxacin and from 45% (*n* = 21; 2019) to 53% (*n* = 35; 2020) for levofloxacin (Figure 9). In contrast, there was an increase in the percentage of strains resistant to ciprofloxacin in 2021, reaching 100% in 2022. The resistance trend for ciprofloxacin is highly statistically significant *p* < 0.001, while for levofloxacin it does not show such significance.

The analysis of antibiotic consumption in the hospital (in DDD/100 patient-days) over the studied 6-year period, showed a significant increase in consumption from 2020 to 2022 (Table 5). The lowest antibiotic consumption was recorded in 2019 (34.7), and the highest in 2022 (62.9). From 2017 to 2019, antibiotic consumption remained at a similar, relatively low level (between 44–34.7 DDD/100 persons) (Table 5).

Despite the highest hospital-wide consumption of antibiotics in the previous year, some drug groups saw a decrease in their consumption (Table 3). This applies, between 2020 and 2022, primarily to quinolones (from 12.7 to 7.3 DDD/100 person) and third-generation cephalosporins, mainly ceftriaxone (from 15.7 to 4.7 DDD/100 person). In contrast, the (already very high) use of penicillins with inhibitors (from 8.3 in 2020 to 25.3 DDD/100 pers. in 2022) and carbapenems (from 2.0 to 2.8 DDD/100 pers.) increased during this period (Table 5).

## 4. Discussion

Bacteria classified as alarm pathogens include *P. aeruginosa* bacilli and *Acinetobacter* spp. resistant to carbapenems, two other drug groups or polymyxins [22]. Unfortunately, the ability of these bacilli to produce carbapenemases is being observed at an increasing rate. In the case of *P. aeruginosa*, the first enzymes capable of inactivating carbapenems were IMP-1 and VIM-1. Nowadays, there are many more, and metal-β-lactamases are considered the most common [23,24]. According to EARS-Net data, the percentage of *P. aeruginosa* strains resistant to carbapenems after 2009 was relatively constant both in Poland and worldwide, ranging from 25 to 23%, respectively [25,26,27,28,29]. Of particular note, however, was 2018, where the percentage of resistant strains in the country was 33% [27]. This coincides with the data of the studied hospital, where the highest percentage of carbapenem-insensitive strains over the past 6 years was recorded in 2018 (30%). In contrast, the rate did not increase from 2019, and even a gradual decrease in the percentage of strains resistant to this group of drugs was noticeable. What is worrisome, however, is that the proportion of non-fermenting *A. baumannii*, which exhibit a wider range of resistance than *Pseudomonas*, is gradually increasing in hospital infections. The number of carbapenemase producers in *Pseudomonas* (4%) in 2021 was, in turn, lower than in *Klebsiella* (11% in 2021) [30]. The situation changed in 2022 the percentage of *Pseudomonas* MBL+ increased to 9%, while the percentage of *Klebsiella pneumoniae* carbapenemase-positive strains increased significantly, up to 40% (unpublished data). The remaining *P. aeruginosa* strains resistant to carbapenems (10% in 2021 and 12% in 2022) are less epidemic strains which do not produce carbapenemases, with a transport type of resistance. These data show that the buildup of carbapenem resistance in *P. aeruginosa* after the pandemic shows a lower rate than that of CPE (carbapenem-resistant *Enterobacterales*). In addition to resistance to β-lactam antibiotics, *P. aeruginosa* have also developed resistance to aminoglycosides [31,32]. In many countries in Europe, as well as worldwide, there has been a decline in the percentage of *Pseudomonas* strains resistant to aminoglycosides after 2009. In China, it decreased to 6.1% in 2017, while in Poland it fluctuated between 20% and 26% in 2017–2019 [26,27,28,33,34]. In the analyzed hospital, the highest number of resistant strains was isolated from infections in 2018, which accounted for about 30%, a result that exceeded the national average. In recent years, the susceptibility of *Pseudomonas* strains to aminoglycosides has definitely improved, and the percentage of resistance has fluctuated in the borders of 7–9%, despite the lack of restrictions on the use of these drugs in therapy. A comparison of data on the susceptibility of *P. aeruginosa* to piperacillin with tazobactam, has revealed that the highest percentage of resistant strains occurred in the analyzed hospital in 2018 (39%), followed by a significant increase in the proportion of susceptible strains. This situation coincided with the national trend, as there was an increase in the percentage of resistant strains in Poland after 2012, which finally reached about 37% in 2018 [27,33]. As of 2019, on the other hand, a decrease in the resistance of these bacteria to piperacillin with tazobactam was observed, shaping up at 20%, both in the hospital studied and nationally, thus approaching the global average of about 13% [26,32]. In the studied hospital, the use of antibiotics with inhibitors has increased significantly in recent years, but this mainly involves amoxicillin with clavulanate (which is inactive against *Pseudomonas*) and results in a decrease in its activity against many hospital pathogens. Piperacillin with tazobactam therefore still remains an active drug combination against *Pseudomonas*.

In Poland, the evolution of *P. aeruginosa* resistance to ceftazidime seems to follow a very similar pattern as resistance to piperacillin with tazobactam. In this case, too, there was a decline in the susceptibility of these bacilli observed already since 2009, reaching in 2018 the highest value in recent years (26.9% of resistant strains) [27,33]. Since 2019, in turn, an increase in the percentage of susceptible strains has been observed [28,29,35,36]. The situation was similar at the analyzed hospital, where the percentage of resistant strains fluctuated between 11% and 27% in 2017–2022, with the highest value recorded in 2018, followed by a twofold decrease in the value of this indicator. In 2019, the national and European averages were 20.1% and 14.1%, respectively, while the percentage of resistant strains in the hospital was only 12%, which was significantly lower, both compared to the national average and the average of EU/EEA countries [28]. This visibly correlates with the tendency in the hospital’s antibiotic policy not to overuse third-generation cephalosporins in therapy. Despite a temporary increase in their use at the start of the pandemic in 2020 to 15.7 DDD/100 patients days (which occurred in many countries), there was a marked decrease in the following years (to 4.7 DDD/100 patient days in 2022). The fact that the rate of resistance to this cephalosporin was low in the described hospital and still does not show an upward trend confirms the usefulness of ceftazidime as a first-line drug in the treatment of infections caused by non-fermenting *P. aeruginosa*.

In turn, resistance to fluoroquinolones, conditioned by mutation in chromosomal genes, leads to inactivation of DNA gyrase. It is very common for this mechanism to coexist with overactive efflux systems, resulting in an almost complete lack of sensitivity of bacterial strains. It is estimated that the most common reason for the increase in resistance to fluoroquinolones is the overuse of these antibiotics in outpatient treatment [37]. The susceptibility of *P. aeruginosa* to quinolones in Asian countries is quite high (86%), while in Poland the percentage of resistant strains over the past 10 years has been relatively constant at around 40% [25,26,27,28,29,33,34,35]. The situation was similar at the analyzed hospital in 2017, while in 2018 there was already a significant increase in the resistance of these bacilli, reaching 61% for levofloxacin. The year also saw record consumption for this group of antibiotics, which may have had an impact on the spread of resistant clones. On the other hand, starting in 2019, as the hospital’s antibiotic policy was programmatically geared toward reducing the use of quinolones, an increase in the susceptibility of Pseudomonas to this group of antibiotics was observed. Despite an increase in the use of antibiotics from this group during the COVID-19 pandemic in 2020 (consumption of 12.7 DDD/100 patient-days), as in the case of third-generation cephalosporins, this consumption declined sharply between 2021 and 2022 (to 7.3 DDD/100 patient-days), without generating an increase in resistance.

Data on the contribution of *A. baumannii* strains to infections indicate that there was a significant increase in the number of *A. baumannii* strains in Asian and European countries during 2020 [29]. This trend was also noticeable in the analyzed hospital, when in 2021 the number of *A. baumannii* strains reached the highest level in the last 6 years, also surpassing the number of resistant *P. aeruginosa* strains. It can be assumed that the increased number of infections caused by these bacilli was due to the higher number of hospitalized patients in severe condition, especially those hospitalized in the ICU, who developed VAP [38,39].

Strains of *A. baumannii* produce carbapenemases belonging to four classes: A, B, C and D according to Ambler [40]. The serine β-lactamases of the B class found in *A. baumannii* are most commonly KPC enzymes and GES family enzymes [41]. According to a CDC report on infections and therapeutics in the United States, *A. baumannii* species exhibiting resistance to carbapenems currently pose the greatest threat to public health [11,42,43]. In 2012 in Poland, the proportion of *A. baumannii* strains resistant to carbapenems was 38%, while in 2017–2019 the proportion has already exceeded 70% and remains at a relatively constant, high level [26,27,28,33]. The CARSS system, on the other hand, reports that in China, about 60% of *Acinetobacter* no longer show sensitivity to this group of antibiotics [34]. At the studied Lower Silesian Specialized Hospital, in 2017–2020, the percentage of carbapenem-resistant strains was below the national average. However, in 2021, there was a sharp increase to almost 80%, most likely due to an increase in the use of this group of antibiotics in therapy. Importantly, during this period of the pandemic there was also a significant increase in the number of *Acinetobacter* strains isolated in infections.

The situation of the analyzed hospital also coincides with the global situation in terms of the significant increase in resistance to carbapenems for *A. baumannii* strains, compared to *K. pneumoniae* [44,45]. Resistance of *A. baumannii* to aminoglycosides in recent years has remained relatively constant at a high level of 73% [28]. In the analyzed hospital in 2021, the percentage of resistant strains also exceeded 70%, while in 2017–2020 it was far below the national average. However, an upward trend for this resistance rate has been observed in recent years.

Fluoroquinolones are a group of antibiotics to which *Acinetobacter* bacilli show the lowest sensitivity among the antimicrobial drugs described above [11,46]. In addition, these bacteria can develop resistance to colistin, which is an antibiotic of last resort. There are two theories about the underlying processes. The first is that there is a mutation causing a complete absence of LPS and, consequently, of lipid A, which is the target site for colistin. The second, on the other hand, suggests that lipid A is present, but in a form altered by mutation of the *pmrA* and/or *pmrB* gene, which affects the lowering of colistin’s affinity for LPS [47]. Increasingly, clinicians are also encountering multidrug-resistant strains, which is probably due to prior antibiotic therapy, very often inappropriately administered [48]. It is believed that for infections caused by *A. baumannii*, monotherapy should not be used, but rather a combination of compounds that would condition protection against the development of resistance. For the time being, polymyxins in combination with carbapenems or tigecycline prevail in the treatment of MDR infections [49]. In Poland, the percentage of resistant strains has reached up to 87% in recent years and remains high [26,27,28,29,35]. In the case of the studied hospital, a similar situation occurred in 2017, 2018 and 2021, while in the 2019–2020 period there was a decrease in the percentage of resistant strains to about 50%, probably due to a reduction in the use of quinolones in 2019. Unfortunately, during the pandemic period there was a renewed increase in the use of antibiotics, including quinolones, in empirical therapy, including in patients with COVID-19, resulting in a huge increase in quinoline resistance for *A. baumannii* strains, of the order of 87%. Again, unfortunately, the programmatically applied reduction in the use of quinolones in the hospital (reduction in consumption in 2021–2022), did not result in an evident reduction in resistance, as in the case of *Pseudomonas* strains. It is true that the resistance of *Acinetobacter* strains to levofloxacin in 2022 fell to 63%, but in the case of ciprofloxacin there was an increase in resistance of up to 100%.

In conclusion, the analyses carried out at the specialized hospital allowed tracing drug resistance among the bacteria most commonly isolated from hospital-acquired infections. Knowledge of pathogen resistance and observations of emerging trends provide the opportunity to correctly apply empirical therapy with much higher effectiveness. In addition, the results made it possible to compare these data with reports of antibiotic consumption in the hospital during the period analyzed. A correlation was observed between the increase in resistance among *A. baumannii* strains and the increase in antibiotic consumption. Undoubtedly, these analyses need to be continued in order to track the development of resistance over a longer period of time. This is important especially after the COVID-19 pandemic, during which an uncontrolled increase in the consumption of broad-spectrum antibiotics undoubtedly contributed to a decline in the susceptibility of the hospital-acquired pathogens studied.

## 5. Conclusions

1. Between 2020 and 2022, the highest consumption of antibiotics in the hospital was observed, most likely due to the COVID-19 pandemic and a greater number of patients in severe condition requiring hospitalization.
2. Unfortunately, during the period under review, the number of multi-resistant strains of *A. baumanii* was increasing successively, which seems to be related to the increased use, especially during the pandemic, of broad-spectrum antibiotics, mainly penicillins with inhibitors, third-generation cephalosporins and carbapenems.
3. The simultaneous programmatic reduction in the use of some antibiotics, such as quinolones and third-generation cephalosporins in the post-pandemic period, seems to have had an impact on the renewed increase in strain susceptibility, especially for *P. aeruginosa*.

## Figures and Tables

**Table 1:** Number of tested strains of particular microorganisms isolated from infections in 2017–2022.

Year/Strains	2017	2018	2019	2020	2021	2022
*Pseudomonas* *aeruginosa*	173	202	158	147	146	149
*Acinetobacter* *baumannii*	123	104	47	66	160	98
Total	296	306	205	213	306	247

**Table 2:** Antibiotics to which the sensitivity of the tested strains was determined.

Strain	Selected Antibiotics
*Pseudomonas aeruginosa*	Gentamicin, amikacin, ciprofloxacin, levofloxacin, meropenem, imipenem, ceftazidime, cefepime, piperacillin/tazobactam
*Acinetobacter baumanii*	Gentamicin, amikacin, ciprofloxacin, levofloxacin, meropenem, imipenem

**Table 3:** Number of all strains isolated from infections in 2017–2022 (1 strain = 1 patient).

Year/Strain	2017	2018	2019	2020	2021	2022
*Escherichia coli*	791	917	851	742	770	822
*Klebsiella pneumoniae*	320	404	303	270	338	473
*Pseudomonas aeruginosa*	173	202	158	147	146	149
*Acinetobacter baumannii*	123	104	47	66	160	98
*Staphylococcus aureus*	532	627	445	365	348	324
*Enterococcus faecalis*	318	284	243	249	350	295
*Enterococcus faecium*	49	97	68	73	87	105
*Streptococcus pneumoniae*	38	61	45	37	30	32
Other microorganisms	23	41	39	47	89	34
Total	2367	2737	2199	1996	2318	2332

**Table 4:** Percentage (%) of isolated *Pseudomonas aeruginosa* and *Acinetobacter baumanii* strains in relation to all strains isolated from infections in 2017–2022.

Year/Strain	2017	2018	2019	2020	2021	2022
*Pseudomonas aeruginosa*	7.3	7.4	7.2	7.4	6.2	6.4
*Acinetobacter baumannii*	5.2	3.8	2.1	3.3	6.9	4.2

**Table 5:** Antibiotic consumption in the hospital during the studied 6-year period: 2017–2022 (in DDD/100 Patient Days).

	Antibiotic Consumption in the Hospital (in DDD/100 Patient Days)													
Year	TET	PES	PES+in.	C II	C III	C IV	KARB	MAK	LINK	AM	CH	GP	POL	Total
2017	1.0	0.8	7.3	9.3	2.6	0.1	0.9	3.4	0.2	0.8	5,7	0.6	0.3	42.4
2018	0.6	0.7	7.2	11.0	3.1	0.2	1.2	0.6	0.6	0.6	9,3	0.9	0.4	44.0
2019	2.4	1.3	8.4	1.4	2.7	0.2	2.4	2.4	0.8	0.7	2,5	1.3	0.2	34.7
2020	0.8	1.4	8.3	2.6	15.7	0.3	2.0	0.6	1.1	1.0	12.7	1.1	0.4	58.3
2021	0.7	3.9	22.3	1.8	7.4	0.2	2.1	0.8	1.0	1.0	8.3	1.4	1.8	60.5
2022	0.7	3.3	25.3	1.8	4.7	0.2	2.8	0.8	1.2	1.5	7.3	1.4	1.6	62.9

TET—tetracyclines, PES—penicillins, PES+in.—penicillins with inhibitors, C II—second-generation cephalosporins, C III—third-generation cephalosporins, C IV—fourth-generation cephalosporins, KARB—carbapenems, MAK—macrolides, AM—aminoglycosides, CH—quinolones, GP—glycopeptides, POL—polymyxins.

## Data Availability

Not applicable.
